# First in man study of [^18^F]fluoro-PEG-folate PET: a novel macrophage imaging technique to visualize rheumatoid arthritis

**DOI:** 10.1038/s41598-020-57841-x

**Published:** 2020-01-23

**Authors:** Nicki J. F. Verweij, Maqsood Yaqub, Stefan T. G. Bruijnen, S. Pieplenbosch, Marieke M. ter Wee, Gerrit Jansen, Qingshou Chen, Philip S. Low, Albert D. Windhorst, Adriaan A. Lammertsma, Otto S. Hoekstra, Alexandre E. Voskuyl, Conny J. van der Laken

**Affiliations:** 1 Amsterdam Rheumatology and immunology Center (ARC), Amsterdam UMC| location VUmc, Amsterdam, the Netherlands; 2 Department of Radiology & Nuclear Medicine, Amsterdam UMC| location VUmc, Amsterdam, The Netherlands; 3 Department of Chemistry and Institute for Drug Discovery, Purdue University, West Lafayette, IN USA

**Keywords:** Molecular imaging, Rheumatoid arthritis

## Abstract

Non-invasive imaging of arthritis activity in rheumatoid arthritis (RA) patients using macrophage PET holds promise for early diagnosis and therapeutic response monitoring. Previously obtained results with macrophage tracer (*R*)-[^11^C]PK11195 were encouraging, but the imaging signal could be further improved by reduction of background uptake. Recently, the novel macrophage tracer [^18^F]fluoro-PEG-folate was developed. This tracer showed excellent targeting of the folate receptor β on activated macrophages in synovial tissue in a preclinical arthritic rat model. We performed three substudies to investigate the biodistribution, potential for imaging arthritis and kinetic properties of [18F]fluoro-PEG-folate in RA patients. Firstly, biodistribution demonstrated fast clearance of [^18^F]fluoro-PEG-folate from heart and blood vessels and no dose limiting uptake in organs. Secondly, [^18^F]fluoro-PEG-folate showed uptake in arthritic joints with significantly lower background and hence significantly higher target-to-background ratios as compared to reference macrophage tracer (*R*)-[^11^C]PK11195. Lastly, dynamic scanning demonstrated fast tracer uptake in affected joints, reaching a plateau after 1 minute, co-existing with a rapid blood clearance. In conclusion, this first in man study demonstrates the potential of [^18^F]fluoro-PEG-folate to image arthritis activity in RA with favourable imaging characteristics of rapid clearance and low background uptake, that allow for detection of inflammatory activity in the whole body.

## Introduction

Rheumatoid arthritis (RA) is a chronic systemic connective tissue disease that primarily affects the joints. Joint inflammation in RA is chronic and may result in progressive destruction of bone and cartilage, eventually leading to loss of function and disability^1^. Joint damage as depicted on conventional X-rays, occurs in 41–60% of patients shortly after onset of RA symptoms^2–4^. Recent international guidelines advise to start treatment as early as possible after the diagnosis of RA in order to prevent further damage and functional disability^5^. Clearly, early initiation of treatment requires early detection of arthritis. In addition, to identify the most optimal treatment amongst many options available, therapy monitoring in an early phase of treatment is required. Advanced imaging techniques can contribute to early diagnosis and therapy monitoring by sensitive detection of synovitis.

A promising molecular imaging technique is positron emission tomography (PET). This non-invasive imaging technique has very high sensitivity (nanomolar level) and its specificity stems from the use of target specific tracers^6^. Moreover, PET can provide quantitative *in vivo* assessments of molecular targets and interactions, and therefore has excellent characteristics for both early diagnosis and monitoring response to therapy^7^. Indeed, it has already been shown that PET can be a valuable *in vivo* tool for assessing RA^8^.

The macrophage in particular is a central target for PET imaging of RA, because of its infiltration in synovium from the early development of RA onwards^9–11^. So far, primarily immunohistochemical methods have been used to identify macrophage infiltration using synovial samples obtained by arthroscopy^12,13^. Although these methods are certainly valuable, they are time-consuming and invasive. As a non-invasive alternative, in RA patients, macrophages have successfully been visualized using PET^14^. Promising results were initially obtained using (*R*)-[^11^C]PK11195 (1-(2-chlorophenyl)-N-methyl-N(1-methyl-propyl)-3-isoquinoline carboxamide). This tracer binds to the 18 kDa translocator protein (TSPO) in the inner mitochondrial membrane of activated macrophages^14,15^. Despite the successful results that were obtained with this macrophage tracer, arthritis imaging by macrophage targeting could be further improved by reduction of background uptake, which was among others present in peri-articular tissues^16^.

This has stimulated the development of alternative macrophage tracers. A potential, emerging target for visualizing macrophages is the β-isoform of the folate receptor (FRβ), a glycosylphosphatidylinositol-anchored cell membrane protein^17^. In contrast to the α-isoform of FR^18–20^, FRβ is selectively expressed on both normal and malignant hematopoietic cells of the myeloid lineage, including monocytes, (tumour-associated) macrophages, and myeloid leukaemia cells^21–23^. Observations that FRβ expression and folate binding are induced during (synovial) macrophage activation in RA^24–27^, and exploiting the property of high binding affinity of folic acid to FRβ^25^, have encouraged the development of folate conjugates as potential macrophage imaging agents for cancer and inflammatory diseases^28,29^. Recently, [^18^F]fluoro-PEG-folate (polyethylene glycol folate) has been proposed as a novel candidate folate-based PET tracer^30^. It proved to be an excellent diagnostic tool for both non-invasive visualization of arthritis and monitoring of therapy response to methotrexate and experimental therapies in arthritic rats^31–33^.

These promising results in a preclinical arthritis model in rat set the stage for the present first in man feasibility study, consisting of three substudies in clinically active RA patients, each applying different scanning procedures: 1. Whole body scan to assess [^18^F]fluoro-PEG-folate tissue distribution and potential dose limiting uptake in organs; 2. Static scans of the hands to assess whether [^18^F]fluoro-PEG-folate PET/computed tomography (CT) can image inflamed hand joints compared with reference macrophage tracer (*R*)-[^11^C]PK11195; and 3. Dynamic scans to assess the kinetic properties of [^18^F]fluoro-PEG-folate in arthritic joints and background.

## Results

### Substudy 1: whole body biodistribution

**Figure 1:**
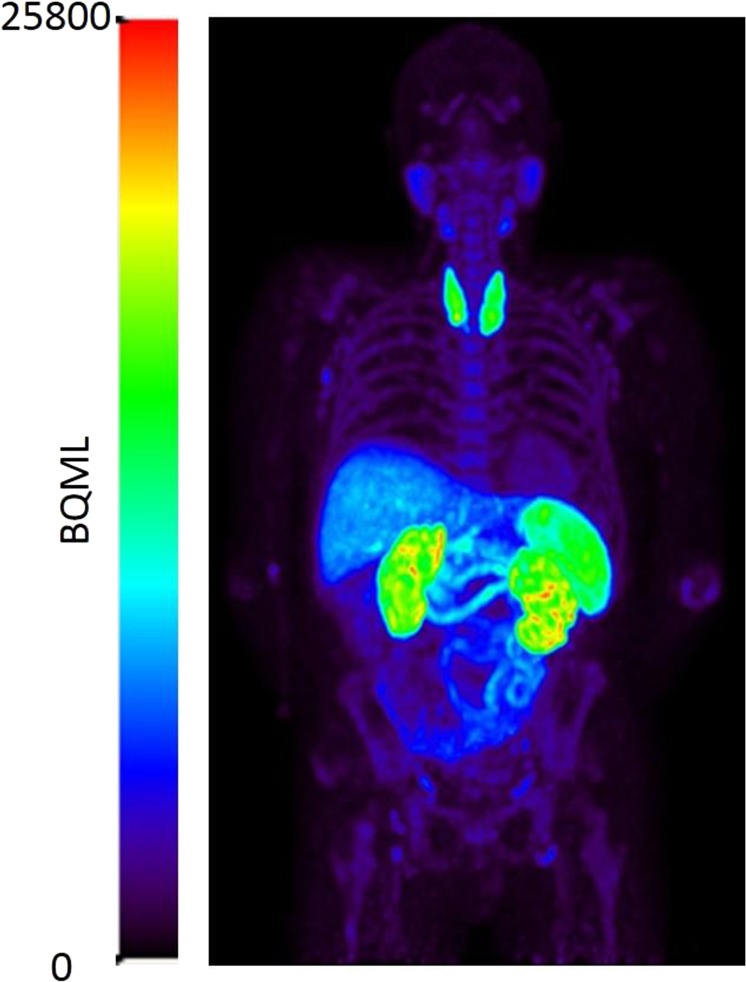
Whole body biodistribution of [^18^F]fluoro-PEG-folate of a RA patient at 1 minute post injection.

Whole body PET (Fig. 1) revealed that [^18^F]fluoro-PEG-folate cleared rapidly from the heart and blood vessels. Low uptake was seen in muscle and bone. Percentage of injected dose (%ID) and organ dose over time are shown in Table 1. The highest organ dose was seen in the liver (3.70E^−03^ mSv/MBq), the thyroid (3.20E^−03^ mSv/MBq) and the spleen (1.99E^−03^ mSv/MBq). The effective dose was calculated to be 0.0168 mSv/MBq.

**Table 1:** %ID per time point (corrected for decay) and total organ dose (in mSv) per organ.

Time post injection	%ID (corrected for decay)				Organ dose (mSv/MBq)
	*1 min*	*37 min*	*72 min*	*107 min*	
Lungs	2.79	2.09	1.69	1.40	1.90E^−03^
Kidneys	9.09	6.88	5.10	3.86	2.16E^−04^
Spleen	6.48	4.97	3.89	3.05	1.99E^−03^
Liver	14.89	13.57	12.07	10.28	3.70E^−03^
Myocardium	0.30	0.22	0.18	0.14	
Thyroid	0.69	0.43	0.31	0.21	3.20E^−03^
Bladder	0.04	0.04	0.02	0.02	1.79E^−05^
Bone marrow	0.11	0.09	0.08	0.06	1.98E^−03^
Parotid glands	0.03	0.01	0.02	0.01	
Aorta descendens	0.00	0.00	0.00	0.00	
Left ventricle	0.00	0.00	0.00	0.00	

### Substudy 2: imaging potential for arthritis in RA patients

#### Clinical data

A total of 9 patients with clinically active RA were included in the comparative substudy between [^18^F]fluoro-PEG-folate and (*R*)-[^11^C]PK11195. The only statistical differences between patients scanned with (*R*)-[^11^C]PK11195 and those scanned with [^18^F]fluoro-PEG-folate was the sex (100% male vs 33% male respectively, *p* = 0.03; Table 2) and number of patients on prednisone (0% vs 67% respectively, *p* = 0.03; Table 2). Patients injected with [^18^F]fluoro-PEG-folate did not experience any side effects.

**Table 2:** Baseline patient demographics, clinical and functional characteristics.

	[^18^F]fluoro-PEG-folate (n = 6)	(R)-[11C]PK11195 (n = 3)	*p*
Male, number (%)	2 (33)	3 (100)	0.03
Age, years (mean ± SD)	60 ± 11	65 ± 13	0.64
Height, cm (mean ± SD)	171 ± 9	175 ± 6	0.49
Weight, kg (mean ± SD)	80 ± 15	88 ± 11	0.50
Disease duration, months (mean ± SD)	73 ± 91	160 ± 156	0.39
IgM RF positivity, number (%)	3 (50)	3 (100)	0.08
Anti-CCP positivity, number (%)	4 (67)	2 (67)	1.00
DAS 28 (mean ± SD)	5.58 ± 1.10	4.79 ± 1.94	0.52
DAS 44 (mean ± SD)	3.82 ± 1.15	2.73 ± 1.24	0.29
44-swollen joint count (median ± IQR)	6.5 ± 8	3	0.80
44-tender joint count (median ± IQR)	9 ± 12	8	0.88
CRP, mg/mL (median ± IQR)	22 ± 72.6	5	0.15
ESR, mm/h (median ± IQR)	36.5 ± 49	20	0.22
VAS disease activity, 0–100 mm (mean ± SD)	52.2 ± 20.9	68 ± 20.5	0.41
DMARD therapy (number (%))	4 (67)	2 (67)	1.00
Oral prednisolone (maximal dosage of 10 mg/day)	4 (67)	0 (0)	0.03
NSAID therapy	2 (33)	1 (33)	1.0

The number of clinically active hand and wrist joints (determined as swollen at the moment of clinical evaluation) ranged between 3 and 16 joints per patient, with a total of 65 swollen joints. The distribution of these joints was 9% wrists, 65% metacarpophalangeal (MCP) joints, and 26% proximal interphalangeal (PIP) joints.

#### Visual interpretation of joint targeting by [^18^F]fluoro-PEG-folate PET

**Figure 2:**
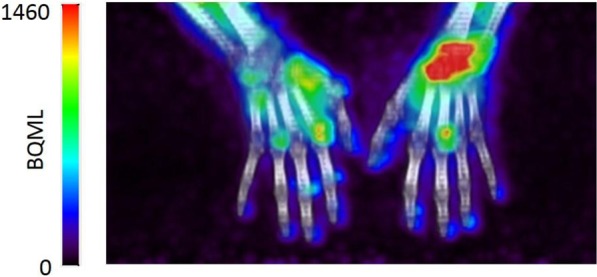
[^18^F]fluoro-PEG-folate uptake in hand/wrist joints of a clinically active RA patient.

[^18^F]fluoro-PEG-folate clearly showed uptake in arthritic joints (Fig. 2). At a patient level, 25 PET positive joints in 6 RA patients were found (range 1–8 per patient). Enhanced uptake was seen in the wrists (24% of all PET positive joints), carpometacarpal (CMC) joints (20%), MCP joints (40%) and PIP joints (16%). The number of PET positive joints did not correlate with any clinical factors including DAS28/44, tender and swollen joint count, nor with serological inflammation markers erythrocyte sedimentation rate (ESR) and C-reactive protein (CRP).

At a joint level, Table 3 summarizes the relationship of clinical and PET findings for the two tracers. Similar true positive values were observed for [^18^F]fluoro-PEG-folate and (*R*)-[^11^C]PK11195, while false positive values were higher for [^18^F]fluoro-PEG-folate. In addition, the number of false negative joints was lower and the number of true negative joints was higher for [^18^F]fluoro-PEG-folate, than for (*R*)-[^11^C]PK11195.

**Table 3:** Comparison of clinical and PET findings between (*R*)-[^11^C]PK11195 and [^18^F]fluoro-PEG-folate.

	Clinically positive		Clinically negative	
	[^18^*F]fluoro-PEG-folate*	*(R)-*[*11C]PK11195*	*[*^18^*F]fluoro-PEG-folate*	*(R)-[11C]PK11195*
PET positive	13 (7.5%)	5 (6.4%)	27 (15.6%)	6 (7.7%)
PET negative	23 (13.3%)	21 (26.9%)	110 (63.6%)	46 (59.0%)

#### Quantitative PET data

At a patient level, median [^18^F]fluoro-PEG-folate uptake (SUV) in all PET positive joints per patient ranged from 0.11 to 0.89. The highest uptake was found in wrists (1.03 ± 0.45) and the lowest in PIP joints (0.23 ± 0.52). No correlation between clinical factors and mean tracer uptake was seen. Four of the patients scanned with [^18^F]fluoro-PEG-folate were using methotrexate in varying dosages (7.5 to 25 mg weekly). No correlation was found between quantitative tracer uptake in joints and methotrexate use or dosage.

Quantitative PET data at joint level are summarized in Table 4.

**Table 4:** Comparison of (*R*)-[^11^C]PK11195 and [^18^F]fluoro-PEG-folate.

	[^18^F]fluoro-PEG-folate	(R)-[11C]PK11195
Joint uptake (SUV)	0.5 ± 0.6	1.3 ± 0.4
Background uptake (SUV)	0.1 ± 0.1	0.8 ± 0.2
T/B ratio	3.5 ± 2.2	1.7 ± 0.6

Although both [^18^F]fluoro-PEG-folate and (*R*)-[^11^C]PK11195 accumulated in arthritic joints, the mean SUV in joints of patients injected with (*R*)-[^11^C]PK11195 was significantly higher than that in joints of patients injected with [^18^F]fluoro-PEG-folate (mean difference: 0.28 [0.19;0.42]; *p* < 0.001). However, [^18^F]fluoro-PEG-folate showed significantly lower background uptake than (*R*)-[^11^C]PK11195 (mean difference: 0.26 [0.24;0.27]; *p* < 0.001), which was more pronounced than the difference in absolute joint uptake. This resulted in significantly higher T/B-ratios of joints visualized with [^18^F]fluoro-PEG-folate (mean difference: 1.11 [0.92;1.34]; *p* < 0.02), which provided clear delineation of the PET positive joints on [^18^F]fluoro-PEG-folate scans, as shown in Fig. 2.

### Substudy 3: kinetic properties

**Figure 3:**
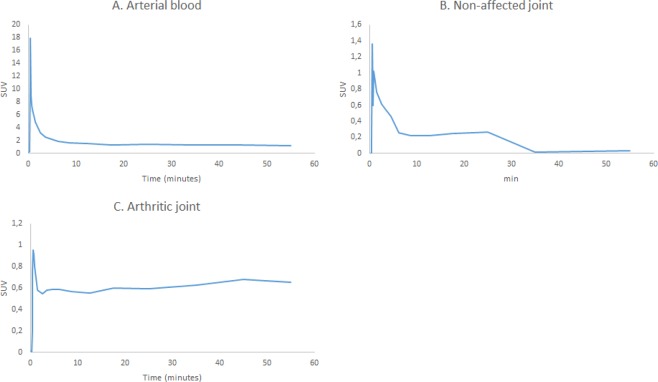
[18F]fluoro-PEG-folate kinetics in arterial blood (**A**), a clinically non-affected joint (**B**) and an arthritic joint (**C**) over time.

For the dynamic substudy, six patients (female 5/6, age 55 ± 8) with clinically active RA^34^ were included. Patients had an average disease duration of 7 ± 6 years and an average DAS28 score of 3.8 ± 1.0. All patients were on steady medication, varying from NSAIDs (50%) to DMARDs (67%) and prednisolone (67%). In each patient, the most clinically affected joint was selected, which was located in a knee (4 patients), foot (1 patient) or wrist (1 patient). On each scan [^18^F]fluoro-PEG-folate showed an increase of uptake in clinically inflamed joints compared with contralateral clinically non-affected joints. SUV and K_i_ showed a linear correlation with a coefficient of determination (R^2^) of 0.78 using arterial blood as an input function in the 2t3k model. K_i_ with arterial blood as input function and K_i_ with venous blood as an input function showed a linear correlation with a R^2^ of 0.94. TAC of tracer in blood showed that [^18^F]fluoro-PEG-folate peaked at 1 minute after injection, followed by rapid clearance from the blood compartment. TAC of arthritic joints reached a plateau in uptake in arthritic joints as soon as approximately 1 minute after injection, followed by stable uptake during at least 60 minutes after injection, while the tracer was washed out from the contralateral clinically non-affected joints (Fig. 3).

## Discussion

This study is the first study in RA patients with the novel tracer [^18^F]fluoro-PEG-folate. The biodistribution was favourable with fast clearance from the bloodstream and low background uptake in major organs. In RA patients, [^18^F]fluoro-PEG-folate showed clear uptake in arthritic joints, with low background uptake in peri-articular bone. Although absolute arthritis uptake was lower, arthritis imaging signal of [^18^F]fluoro-PEG-folate was improved as compared with the reference macrophage tracer (*R*)-[^11^C]PK11195, due to significantly higher T/B-ratios as a consequence of even more pronounced reduction of background. In addition, dynamic scanning with [^18^F]fluoro-PEG-folate demonstrated fast uptake in arthritic joints, which remained stable over time.

The [^18^F]fluoro-PEG-folate biodistribution data were in line with previous preclinical studies;^30–33,35^. The effective dose of 0.0168 mSv/MBq is lower than similar [^18^F] tracers such as fludeoxyglucose (FDG), which is currently the main tracer used in clinical practice for imaging of inflammation. Uptake in liver and spleen of arthritic patients can be explained, at least in part, by hepatobiliary secretion of the tracer and possible increased macrophage infiltration in these organs reflecting systemic inflammatory effects. Uptake in the kidneys may be due to both renal clearance and binding to locally present folate receptor α (FRα), as [^18^F]fluoro-PEG-folate binds to both FRα and FRβ with comparable affinities^25,30^. Uptake in the thyroid can also be ascribed to locally present FRα^36,37^. The folate PET data in RA patients also corresponded with imaging data using [^99m^Tc]folate, a single-photon emission computed tomography (SPECT) tracer^38^. Notably, PET imaging is superior to SPECT with regards to contrast and spatial resolution, but also provides the ability to use tracers with a lower half-life and thus patients will be exposed to a lower radiation dose^39^.

Compared with (*R*)-[^11^C]PK11195, uptake of [^18^F]fluoro-PEG-folate in joints was 2 to 3 fold lower. However, since background uptake for the folate tracer was more reduced (4 fold lower), a significantly higher target to background ratio and consequently superior visual identification of affected joints was obtained. In addition, low activity in the blood pool, muscles and bone makes [^18^F]fluoro-PEG-folate potentially applicable for imaging of (systemic) inflammation in other organs and arthritis visualization in the whole body. [^18^F]fluoro-PEG-folate imaging seemed to correspond better with clinical outcomes than (*R*)-[^11^C]PK11195 imaging, with higher true positive and true negative values. An exception was the higher number of false positives found in patients scanned with [^18^F]fluoro-PEG-folate. This may, however, also point to better detection of subclinical arthritis. Indeed, uptake of the aforementioned folate-targeted ^99m^Tc SPECT imaging agent has been reported in subclinical RA. In addition, uptake in osteoarthritic joints has also been described and may have been another explanation for false positive uptake in some joints^40^.

Initial findings of full kinetic analysis showed that a 2T3k model was optimal for fitting [^18^F]fluoro-PEG-folate kinetics. A good correlation between SUV and K_i_ was found, demonstrating the validity of SUV as an outcome measurement. The fast time to plateau of approximately 1 minute and steady state of tracer uptake in arthritic joints throughout 60 minutes of scanning is different to previous findings of macrophage tracers in RA^41^. These characteristics provide broad opportunities for simplified (i.e. static) scanning protocols, as a broad time range appears suitable for scanning and initiation of scanning shortly after injection. However, the low sample size dictates the need for further kinetic analyses in a larger cohort, in particular for investigation of therapeutic effects on tracer kinetics when used for therapy monitoring.

Four out of nine patients used methotrexate in varying dosages (7.5 to 25 mg weekly). To prevent potential blocking of [^18^F]fluoro-PEG-folate binding to FRβ by methotrexate, all patients were instructed to keep a minimum of 7 days between methotrexate intake and scanning. With this interval it is unlikely that tracer uptake in joints is lowered due to FRβ occupancy by methotrexate, as residual plasma levels of methotrexate were very low (<10 nanomolar) at that stage^31^. Moreover, [^18^F]fluoro-PEG-folate binding affinity towards FRβ outweighs methotrexate by at least 2–3 orders of magnitude^25,30^. This is illustrated by one patient who mistakenly took methotrexate on the day before the scan. Even in this patient [^18^F]fluoro-PEG-folate uptake in joints was observed within the same order of magnitude as for the other patients, although some level of suppression of tracer uptake in the joints of this patient cannot be ruled out. This suggests that methotrexate treatment does not significantly interfere with [^18^F]fluoro-PEG-folate PET imaging of arthritis activity, which is relevant for clinical applicability in RA patients. Furthermore, no correlation was found between quantitative tracer uptake in joints and methotrexate use or dosage. To prevent any possible FR competition, however, it is recommended to perform [^18^F]fluoro-PEG-folate PET scans at least 7 days after the last methotrexate intake.

There are some limitations to this study. First, sample sizes were small due to the clinical proof of concept character of the study. Nevertheless, feasibility of [^18^F]fluoro-PEG-folate PET to image arthritis in RA could be clearly demonstrated. Secondly, differences in quantitative uptake between tracers were statistically significant, even after adjustment for the multilevel structure of the data. Thirdly, [^18^F]fluoro-PEG-folate and (*R*)-[^11^C]PK11195 scans were not acquired in the same patients, so no true head to head comparison could be performed. To allow comparison in different patients, patients in both groups were selected carefully for similar clinical disease activity. Although subgroups receiving different tracers were too small to allow for correction for confounders as treatment differences, obtained data did not point at any effect of treatment type/dose on tracer uptake in clinical arthritis, since all patients had active clinical arthritis despite treatment. Lack of significant effect of immunosuppressive treatment of tracer uptake in clinically active arthritis is also supported by previous study findings by our group. These studies showed that (*R*)-[^11^C]PK11195 uptake in arthritic joints was related to the level of clinical arthritis activity but not to presence and type of immunosuppressive treatment^14,42,43^. For folate receptor targeting, data in synovial fluid of clinically active arthritic joints of RA patients demonstrated similarly upregulated levels of FRβ expression on macrophages (as compared to healthy controls) independent of presence and type of immunosuppressive treatment (own unpublished data). Additionally, it has been demonstrated that in clinically active RA, FRβ is increased on activated macrophages, while this is not present on non-activated macrophages^24,44^. Finally, only small fields of view were scanned per patient, leaving other, potentially (sub)clinically affected joints, outside the field of view of the PET scanner. Although images of hands may well represent inflammatory status of other joints^45,46^, future studies should also include whole body images to investigate the performance of [^18^F]fluoro-PEG-folate for visualization of arthritis activity in other joints.

The potential of macrophage PET imaging for clinical application in RA has previously been established^8,14,16^. The favourable imaging characteristics for visualization of (subclinical) arthritis activity by macrophage targeting makes [^18^F]fluoro-PEG-folate PET promising for whole body imaging of arthritis activity in RA. The use of a F-18 radio-isotope with a half-life of 110 minutes allows for central synthesis and regional distribution to other medical centers. Potential clinical applications are early diagnosis of RA and early assessment of therapeutic efficacy of FRβ-targeted macrophage therapies, which can support development of personalized medicine^29^. Moreover, fast clearance from the blood pool makes [^18^F]fluoro-PEG-folate interesting for imaging systemic inflammation in RA and potentially other diseases (such as atherosclerosis or vasculitis), which can be depicted in one whole body imaging session.

## Methods

### Patients

All methods were performed in accordance with the relevant guidelines and regulations. Ethical approval was obtained from the Medical Ethics Review Committee of the VU Medical Center. All patients gave written informed consent prior to inclusion.

In the first substudy, one patient (male, age 70) with clinically active RA based on American College of Rheumatology/European League Against Rheumatism (ACR/EULAR) criteria was included for [^18^F]fluoro-PEG-folate whole body scanning^34^. This patient had a disease duration of 18 years, a DAS44 (disease activity based on 44 joints) score of 2.9 (4 tender joints, 8 swollen joints) and received leflunomide treatment (10 mg daily)^47^.

In the second substudy investigating the arthritis imaging potential of [^18^F]fluoro-PEG-folate PET/CT, 9 patients (female 4/9, age 62 ± 12) with clinically active RA^34^ were included between November 2013 and July 2016. Patients (>18 years) were included if they had at least two clinically inflamed hand and/or wrist joints (defined as swollen at clinical evaluation). In addition, they had to be on stable treatment, defined as a period of one month for non-steroidal anti-inflammatory drugs (NSAIDs) and a period of three months for disease modifying anti rheumatic drugs (DMARDs) and/or biologicals. Treatment could include oral corticosteroids with a maximum dose of 10 mg per day. Patients were excluded from the study if they had been exposed to radioactivity above 5 mSv for research purposes in the last year, had taken experimental drugs in the previous 3 months, or if they were either pregnant or breast-feeding. Patients were asked to discontinue benzodiazepine treatment (if used) for at least 10 days prior to (*R*)-[^11^C]PK11195 scanning for potential competition of binding to TSPO receptors.

Following preliminary analyses to assess the value of [^18^F]fluoro-PEG-folate, a third substudy to assess the kinetic properties through dynamic imaging of [^18^F]fluoro-PEG-folate was performed. For this study, six patients (female 5/6, age 55 ± 8) with clinically active RA^34^ were included. The inclusion and exclusion criteria from the second substudy were applied, with addition of anemia (hemoglobin <6.0 mmol/L) and renal insufficiency (GFR <30 mL/min/1.73 m2) as exclusion criteria. Patients were examined to determine the clinically most inflamed joint, which was determined to be the region of interest for dynamic scanning.

### Clinical assessment

Following inclusion, demographical and clinical data were collected. In order to evaluate clinical activity, physical examination of 44 joints was performed and a blood sample was taken to assess the presence of inflammation markers. Patients who were treated with methotrexate were asked to take the last dose no later than 7 days before each scan.

### PET/CT imaging

PET tracers were produced according to Good Manufacturing Practice (GMP). (*R*)-[^11^C]PK11195 is in routine use for clinical studies and was synthesized as described previously^14^. [^18^F]fluoro-PEG-folate was synthesized in a two-step procedure as described previously^30^. Preceding this human application, a single-dose two-week toxicity study in Sprague-dawley rats (executed at Advinus Bangalor, India) did not reveal any safety issues following administration of 0.28 mg/kg fluoro-PEG-folate (data on file).

### Static scanning

Static PET/CT scans were performed using either a Gemini TF-64 or Ingenuity TF-128 PET/CT scanner (Philips Healthcare, Best, the Netherlands). No fasting or premedication was required. Patients received two venous cannulas, one for tracer injection and the other for blood sampling during scanning.

The first in man whole body scans were performed after intravenous injection of a microdose (72.8 MBq) of [^18^F]fluoro-PEG-folate, and were acquired at t = 1, 37, 72 and 107 minutes post injection. Whole body scans were analysed to assess whether there were any potential dose limiting abnormalities in the biodistribution of [^18^F]fluoro-PEG-folate. Notably, this patient was not included for arthritis imaging of the hands as outlined below.

Patients included in the second substudy were injected with either 194 ± 6 MBq [^18^F]fluoro-PEG-folate (n = 6) or 417 ± 20 MBq (*R*)-[^11^C]PK11195 (n = 3). After each injection, the intravenous catheter was flushed with 20 mL NaCl 0.9%. Patients were scanned supine, with the ventral side of the hands on the upper legs. The hands were placed in a special vacuum pouch for immobilisation. Static emission scans of two fields of view (FOVs) comprising hands and wrists were obtained. (*R*)-[^11^C]PK11195 scans were acquired for 5 minutes per FOV, staring 20 minutes after injection. Four repeat [^18^F]fluoro-PEG-folate scans were acquired for 4 minutes per bed position, starting 10, 18, 26 and 34 minutes post injection, respectively. In case of [^18^F]fluoro-PEG-folate, blood samples were withdrawn after each emission scan. The PET scans were preceded by a low dose 35 mAs CT scan for attenuation correction and anatomical localization of the PET signal. Patients were scanned for a maximum total time of 40 minutes.

### Dynamic scanning

During the third substudy, dynamic PET/CT scans of previously determined region of interest was performed using an Ingenuity TF-128 PET/CT scanner (Philips Healthcare, Best, the Netherlands). In line with static scanning, no fasting or premedication was required and two venous cannulas were placed. In addition, an arterial cannula was placed in the radial artery by an experienced anaesthesiologist under local anaesthesia. Patients were scanned supine, with the ventral side of the hands on the upper legs in a special vacuum pouch for immobilisation. Prior to dynamic scanning, a low dose 35 mAs CT scan was performed for attenuation correction and anatomical localization of the PET signal. A 1-hr dynamic [^18^F]fluoro-PEG-folate scan of the region of interest was performed after injection of a bolus of 192 ± 10 MBq [^18^F]fluoro-PEG-folate. Continuous arterial sampling was performed to monitor the arterial radioactivity concentration^48^. Manual venous and arterial samples were drawn at 5, 10, 20, 35 and 60 minutes post injection, after which the line was flushed with heparinised saline. After the dynamic scan, a static scan preceded by a low dose CT scan was performed of the same area. This static scan was followed by one venous sample, at approximately 70 minutes post injection.

### Image analysis

All scan data were corrected for decay, scatter, random coincidences and photon attenuation using established procedures^49^. Reconstructed images were transferred to off‐line workstations for further analysis. The dynamic scans were reconstructed into 19 frames with progressively increasing frame durations (1 × 10, 4 × 5, 2 × 0, 2 × 20, 4 × 30, 4 × 60, 1 × 150 and 4 × 300 seconds).

For the patient in the first study, scans were analysed with respect to biodistribution as function of time. Uptake was measured in major organs (kidneys, liver, lungs, spleen and heart) and additional regions of interest (e.g. thyroid and bladder). Tracer uptake was corrected for patient weight and amount of injected tracer to calculate the percentage of injected dose (%ID), organ dose and effective dose, using OLINDA/EXM^50^.

In the second substudy, visual assessment of tracer uptake in the joints was performed by two trained observers (NV and SB), looking at the wrists, metacarpophalangeal (MCP) and proximal interphalangeal (PIP) joints (n = 22 per patient). Tracer uptake in the joints was quantified by drawing volumes of interest (VOIs) over joints that were visually determined as positive for enhanced tracer uptake, in order to calculate standardized uptake values (SUVs). VOIs were drawn using in-house developed software with the low-dose CT as anatomical reference. To determine a background (reference) value, a standardized spherical VOI was drawn on a non-affected metacarpal bone (outside the joint region). Size and anatomical position of VOIs were determined in consensus by the two observers. SUVs were calculated by dividing the decay corrected radioactivity concentration in each VOI by the injected radioactivity, and normalizing it to body weight. The value used to represent tracer uptake was SUV_peak_, defined as the highest average uptake within a sphere of 1.2 mL^51,52^. The background value provided by the VOI in non-affected metacarpal bone was used to calculate target-to-background (T/B) ratios. As the coefficient of variation was low (median 0.04; interquartile range 0.03–0.06), the average SUV and T/B ratio for each joint was used.

In the third substudy, a VOI was drawn over the previously determined most clinically inflamed joint. To determine a background value, a VOI of identical size and shape to the affected joint was drawn on the contralateral clinically non-affected joint. VOIs were projected onto the dynamic PET image sequences in order to determine regional time activity curves (TACs). The blood collected through venous and (continuous) arterial sampling was used to determine radioactivity concentration ratios and metabolites. In house build software in Matlab (version R2017B, Mathworks, USA) and IDL (version 8.5.1, Exelis Visual Information Solutions, USA) was used for pre-processing VOI definition and pharmacokinetic analysis. Kinetic modelling using several models was applied, of which an irreversible two tissue compartment kinetic (2T3k) model showed the best fit. Using metabolite corrected plasma input curves, the 2T3k model with the fractional blood volume incorporated was used to derive the influx rate constant (K_i_). The agreement between K_i_ and SUV was assessed to determine the validity of SUV.

### Statistical analysis

Continuous variables with Gaussian distribution were summarized as mean ± standard deviation (SD) and 95% confidence interval (CI), and variables that were non-normally distributed were summarized as median and interquartile rang (IQR). SPSS version 22.0 software (SPSS, Chicago IL, USA) was used to assess the distribution of both clinical and PET data. Visual interpretation of the PET/CT data was described using descriptive statistics. To take into account the multilevel structure of the data and to determine differences in scores between the two tracers, two level linear regression analyses with random intercepts and slopes were performed. The first level was defined at the patient level, the second at the joint level. Skewed variables were log-transformed to obtain normality before entering them into regression analyses. Outcome variables were the target score, the background score and the target to background score. The linear correlations between results from the dynamic scans were analysed using a Pearson correlation. Multilevel analyses were performed using MLWin 2.28 software (Centre for Multilevel Modelling, University of Bristol, London, UK). A P-value smaller than 0.05 was considered to be significant.

## Data Availability

The datasets used and/or analysed during the current study are available from the corresponding author on reasonable request.
